# *Irx3/5* Null Deletion in Mice Blocks Cochlea‐Saccule Segregation and Disrupts the Auditory Tonotopic Map

**DOI:** 10.1002/cne.70008

**Published:** 2024-12-10

**Authors:** Bernd Fritzsch, Xin Weng, Ebenezer N. Yamoah, Tianli Qin, Chi‐Chung Hui, Laura Lebrón‐Mora, Gabriela Pavlinkova, Mai Har Sham

**Affiliations:** ^1^ Department of Neurological Sciences University of Nebraska Medical Center Omaha Nebraska USA; ^2^ School of Biomedical Sciences The Chinese University of Hong Kong Shatin Hong Kong SAR China; ^3^ Department of Translational Neuroscience College of Medicine University of Arizona Phoenix Arizona USA; ^4^ Program in Developmental & Stem Cell Biology The Hospital for Sick Children Toronto Ontario Canada; ^5^ Laboratory of Molecular Pathogenetics Institute of Biotechnology CAS Vestec Czechia

**Keywords:** brainstem, cochlea, development, tectorial membrane

## Abstract

A gene cadre orchestrates the normal development of sensory and non‐sensory cells in the inner ear, segregating the cochlea with a distinct tonotopic sound frequency map, similar brain projection, and five vestibular end‐organs. However, the role of genes driving the ear development is largely unknown. Here, we show double deletion of the Iroquois homeobox 3 and 5 transcription factors (*Irx3/5* DKO) leads to the fusion of the saccule and the cochlear base. The overlying otoconia and tectorial membranes are absent in the *Irx3/5* DKO inner ear, and the primary auditory neurons project fibers to both the saccule and cochlear hair cells. The central neuronal projections from the cochlear apex‐base contour are not fully segregated into a dorsal and ventral innervation in the *Irx3/5* DKO cochlear nucleus, obliterating the characteristic tonotopic auditory map. Additionally, *Irx3/5* deletion reveals a pronounced cochlear‐apex‐vestibular “vestibular‐cochlear” nerve (VCN) bilateral connection that is less noticeable in wild‐type control mice. Moreover, the incomplete segregation of apex and base projections that expands fibers to connect with vestibular nuclei. The results suggest the mammalian cochlear apex is a derived lagena reminiscent of sarcopterygians. Thus, *Irx3 and 5* are potential evolutionary branch‐point genes necessary for balance‐sound segregation, which fused into a saccule‐cochlea organization.

## Graphical abstract

Deletion mutant of *Irx3*/*5* shows the cochlear base fused with the saccule, whereas in wild‐type mice, the saccule is separated from the base by the ductus reuniens. *Irx3* and *Irx5* are potential evolutionary branch‐point genes necessary for balance‐sound segregation.

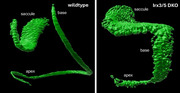

## 1 Introduction

Our gravitational sense and sound perception are detected by specialized sensory end‐organs in the inner ear that evolved from the macular‐cupular vestibule to the cochlear‐tectorial membrane (TM) systems in sarcopterygians (Fritzsch, Schultze, and Elliott 2023; Goodyear et al. 2017). Emerging results show that genetic alterations result in abnormal cochlea and hair cells (HCs), while the vestibular system remains virtually normal (Driver and Kelley 2020; Fritzsch et al. 2013). For example, the loss of *Pax2* leads to the absence of cochlear HCs and spiral ganglion neurons (SGNs) (Bouchard et al. 2010; Burton et al. 2004). The loss of *Lmx1a* converts the base of the organ of Corti into a mosaic of vestibular and cochlear apical HCs (Nichols et al. 2008), and vestibular HCs are reduced but enlarged (Mann et al. 2017; Steffes et al. 2012). Like with *Pax2* knockout (KO), *Lmx1a/b* double KO results in the loss of all cochlear HCs, neurons, and the cochlear nuclei (CN) (Chizhikov et al. 2021). Similarly, the loss of the transcription factor, *Gata3* (Duncan and Fritzsch 2013), and the signaling molecule *Shh* (Bok et al. 2013; Muthu et al. 2019) results in the loss of all cochlear neurosensory development. Other gene alterations affect the morphology and cochlear HC numbers (Pauley, Lai, and Fritzsch 2006; Sun and Liu 2023). Additional genes that are needed for normal neurosensory development are *Eya1* (Xu et al. 2021) and *Sox2* (Dvorakova et al. 2020), which are upstream of *Neurog1* (Elliott et al. 2021) and interact with *Foxg1* (Pyott et al. 2024) and *Tbx1‐3* (Bi et al. 2024; Kaiser et al. 2022). The role of the Iroquois genes (*Irx*) in ear development is incompletely understood in mammals (Leung et al. 2019; Liu et al. 2017).

*Irx* encodes a family of six genes conserved across vertebrates and invertebrates (Tan et al. 2020). The six Iroquois‐related homeobox genes form two clusters, *IrxA* and *IrxB*. *Irx3, Irx5*, and *Irx6* belong to the IrxB cluster and play a role in limbs, adiposis, kidney, oocytes, and brain development (Li et al. 2014; Newton et al. 2022; Tan et al. 2020; Tao et al. 2020). IRX proteins regulate developmental transcription factors. *Irx3* and *Irx5* are positioned close to each other in mouse chromosome 8 and can be deleted combined (Tao et al. 2020). *Irx3/5* double KO (DKO) has a loss of specific limb areas (tibia, digit 1), while the development of all other digits is unaffected, indicating role specificity in limb segments (Li et al. 2014; Tao et al. 2020). *Irx3/5* DKO is required before *Shh, Gli3*, and *Hand2* affect mesenchymal cell movements (Dou, Son, and Hui 2021). Shared protein domains of *Irx3/5* interact with *Eya1, Sox2, Shh, Foxg1*, and *Gata3* in the ear, and similar interactions may occur with *Irx1/2/4* (IrxA) and *Irx6* (IrxB), which have similar expression patterns (Kerner et al. 2009). Moreover, unique interactions of *Shh* and *Irx3* define the position of motoneurons (Exelby et al. 2021). In addition, mutations in *IRX5* in patients show Hamamy syndrome, a severe clinical phenotype, including hearing defects (Farmer et al. 2021).

To understand the role *Irx3/5* plays in ear development, we analyzed two allelic mouse mutants, *Irx3* ^−/−^ and *Irx5* ^−/−^ (*Irx3/5* DKO). *Irx3* and *Irx5* are expressed in the otic placode from E8.5 (Liu et al. 2017). In the E9.5‐E10.5 otic vesicle, *Irx* genes share a broad expression domain in the non‐sensory epithelium and periotic mesenchyme. *Irx3/5* DKO mutant showed morphological abnormalities, characterized by a shortened cochlear duct and fusion of the basal turn of the cochlea with the saccule, consistent with the expression pattern of *Irx5* in the saccule and basal turn (Leung et al. 2019). Instead of a single and three rows of inner (IHC) and outer HCs (OHC), respectively, the basal turn of the greater epithelial ridge [GER; otherwise known as Kölliker's organ (Driver and Kelley 2020)] in *Irx3/5* DKO is transformed into ten rows or more of vestibular‐like HCs, showing opposing polarity as saccular HCs. In addition, non‐sensory cell development is disrupted, resulting in an undifferentiated flat epithelium without otoconia and the TM in the inner ear of *Irx3/5* DKO. Central projections show an overlap of cochlear central projections from the SGNs in the base and apex. A short apical central projection resembles the lagena found in most sarcopterygians (Fritzsch, Schultze, and Elliott 2023).

## 2 Materials and Methods

All animal experiments were performed according to the United States Animal Welfare Act and the use of laboratory animals for research under established guidelines, supervision, and approved protocols by the Animal Experimentation Ethics Committee of the Chinese University of Hong Kong (20‐185‐GRF). The proximity of *Irx3* and *Irx5*, adjacent to chromosome 8, allows the deleting of both genes. We used available *Irx3* ^−/−^ and *Irx5* ^−/−^ mutant mice (Li et al. 2014; Liu et al. 2017). We refer to them as *Irx3/5* DKO (double null mutants). Mice are dead at ∼E17.5, and analyses can be studied from E15.5 to E16.5. The genotype was confirmed by PCR analysis on tail DNA with specific primers.

*Irx3* forward: GAGTTGGCCGCCTCTGGGTCCCTATCCAAT,
*Irx3* reverse: CCCTCTCTCCCGGGTTTCTCTGGCTCTTAC,
LacZ_forward: ACCTCCCACACCTCCCCCTGAACCTGAAAC;
*Irx5*_forward: GGTCCCAAGGGCCAGAATCAGAATTGGGG,
*Irx5*_reverse: GCATTCTTCCGGTACGCGGGGTCCCCATA,
PGK_reverse: CCGGTGGATGTGGAATGTGTGCGAGGCCA.

Embryos were fixed in 4% PFA with 300 mM sucrose to reduce the swelling of the whole head (Schmidt and Fritzsch 2019). Fixed embryos were shipped in 0.4% PFA with 300 mM sucrose to continue processing. All animal experiments were performed according to the United States Animal Welfare Act and the use of laboratory animals for research under established guidelines, supervision, and approved protocols by the Animal Experimentation Ethics Committee of the Chinese University of Hong Kong (20‐185‐GRF).

### 2.1 Antibody Characterization

Tissues were prepared and permeabilized, and non‐specific binding was blocked with 30% goat with 0.3% Triton‐x‐100 in 1x PBS for 24 hours at 4°C. Next, we incubated in anti‐tubulin and anti‐filament, anti‐myosin VIIa, and anti‐NeuN diluted with 1% serum (goat) with 0.1% Triton X‐100 in 1× PBS overnight at 4°C. Secondary antibodies were diluted 1:1000, using Alexa 488, 546, and 647 for 2 h. In addition, we counterstained with DAPI for nuclear labeling (Life Technology; 405 excitation). Dissected ears were imaged with either Leica LS8 or Zeiss 700.

Specifically, we used a primary monoclonal anti‐acetylated tubulin that was produced in mouse and can be used for immunohistochemistry against mouse (Sigma‐Aldrich Cat# T7451, RRID:AB_609894). A secondary we used was a goat anti‐mouse IgG Alexa Fluor 647 (A‐21235, RRID:AB_2535804) to label the primary labeling. In addition, we used the well‐characterized primary polyclonal rabbit anti‐myosin VIIa (Proteus Biosciences Cat# 25–6790, RRID:AB_10015251). As a secondary labeling, we used a goat anti‐rabbit IgG Alexa Fluor 488 (Invitrogen, A‐11008, RRID:AB_143165). Also, we used a recombinant monoclonal rabbit against anti‐NeuN (Cell Signaling Technology Cat# 12943, RRID:AB_2630395). As a secondary, we used goat anti‐rabbit IgG Alexa Fluor 546 (Invitrogen, A‐11010, RRID:AB_2534077). We used NeuN in several cases to label neurons selectively. Additional information was used with a polyclonal chicken anti‐neurofilament (Millipore Cat# AB5539, RRID:AB_11212161). The antibodies were determined and consolidated by the curator on 8/2018. Labeling the primary antibodies, we used goat anti‐chicken IgY Alexa Fluor 488 (A‐11039, RRID:AB_2534096).

For specific labeling of the TM, we used two distinct antibodies. The previously used anti‐Pendrin was a polyclonal antibody in rabbit. Meanwhile, the same antibody is now generated against goat (R and D Systems Cat# BAF2050, RRID:AB_2143488). We used the remaining secondary with goat anti‐rabbit IgG Alexa Fluor 488 (A‐11008, RRID:AB_143165). For details of using the anti‐Pendrin, it was described by Koo et al. (2009). We also used a polyclonal rabbit antibody against Collagen II (Thermo Fisher Scientific Cat# PA1‐26206, RRID:AB_779883). This anti‐collagen has been used in many applications that labels selectively the Col II in the ear. As a secondary antibody, we used goat anti‐rabbit IgG Alexa Fluor 546 (A‐11010, RRID:AB_2534077) to allow double and distinct labeling of Pendrin and Col II.

### 2.2 Dye Tracing

Three‐color labeling excited at 488, 565, and 636 nm wavelengths was used to trace neuronal projection from the brainstem to the inner ear end‐organs by inserting a dye into the ventral base near rhombomere 4 to label facial neurons and the inner ear efferents (IEE). Likewise, we inserted the apex, base, saccule, and utricle (including the anterior and horizontal canal) with three distinct dyes. The dye was allowed to diffuse at 36°C for about 2–3 days.

Ears were prepared and mounted as whole mounts to verify the central projection after dye injection to the brainstem or the ear to image the distribution and size of vestibular neurons. In addition, we imaged the ears as whole mounts and dissected the cochlea, saccule, and utricle, which develop a unique interconnection from the utricle to the apex.

The brainstem was imaged first as a whole mount to present the topology of the cochlear afferents after the dye was inserted into the inner ear. Next, tissue was briefly blotted with paper (Spilfyter, Sigma‐Aldrich #Z558591), embedded in 3% agarose (Millipore‐Sigma #A0576) using Tissue‐Tek disposable Cryomolds (Sakura Finetek #4566 and #4557), and then placed on a flat dry ice block to set. When hardened, the block/sample was trimmed, mounted, and re‐embedded in 3% agarose on the stage of a Compresstome VF‐700 microtome (Precisionary Instruments Inc.). The samples were sectioned in the coronal plane at 100‐µm steps, collected in 0.4% PFA, and temporarily stored at 4°C. Sections were placed in glycerol on a slide, DAPI was added to label the cell nuclei, covered by slides, and imaged using either the Leica LS 8 (10x with a 0.5 NA; 20× with a 0.8; 63× with a 1.4 NA) or the Zeiss 700 (10× with a 0.45 NA; 20× with 0.8 NA; 40× with 1.35 NA). Images were labeled and organized into the figure plates using CorelDraw software.

### 2.3 Scanning Electron Microscopy (*SEM*)

Mice inner ears (E16.5) were postfixed in 2.5% glutaraldehyde followed by 1.0% OsO_4_ fixation. The cochlear TM was removed with fine scissors in control mice. Note that only control cochlear samples were covered with the TM. For *Irx3/5* DKO mouse samples, neither otoconia nor canal cristae were noted. *SEM* preparations were critical point dried, sputter coated, and viewed with a Hitachi S‐4800 *SEM*. Cochleae of age‐matched controls and mutant littermates were processed together but differed in being either left or right ear for easy identification.

### 2.4 3D Light‐Sheet Fluorescent Microscopy (LFSM)

The inner ears were microdissected from WT and *Irx3/5* DKO at E16.5. We used an advanced CUBIC protocol (Susaki et al. 2015) for tissue clearing to enable efficient imaging by light‐sheet microscopy. Briefly, the microdissected inner ears were fixed in 4% PFA for 1 h, washed with PBS, and incubated in a clearing solution, Cubic 1, for 7 days at 37°C. Before immunolabeling, samples were washed in PBT (0.5% Triton‐X in PBS) 4× for 30 min. Samples were immunolabeled using anti‐NeuN (a nuclear marker of differentiated neurons), anti‐Myo7a (HC marker) antibodies, and anti‐beta‐III Tubulin (TUJ‐1, innervation). Samples were stored at room temperature before imaging. The Zeiss Lightsheet Z.1 microscope with illumination objective lightsheet Z.1 5×/0.1 and Z.1 5×/0.16 was used for imaging at the Light Microscopy Core Facility of the Institute of Molecular Genetics of the Czech Academy of Sciences. IMARIS software v8.1.1 (Bitplane AG, CA, USA) was used for image processing.

## 3 Results

### 3.1 HCs in the Cochlear Base of *Irx3/5* DKO Show a Gradual Transition From Vestibular to Cochlear Phenotypes

**FIGURE 1:**
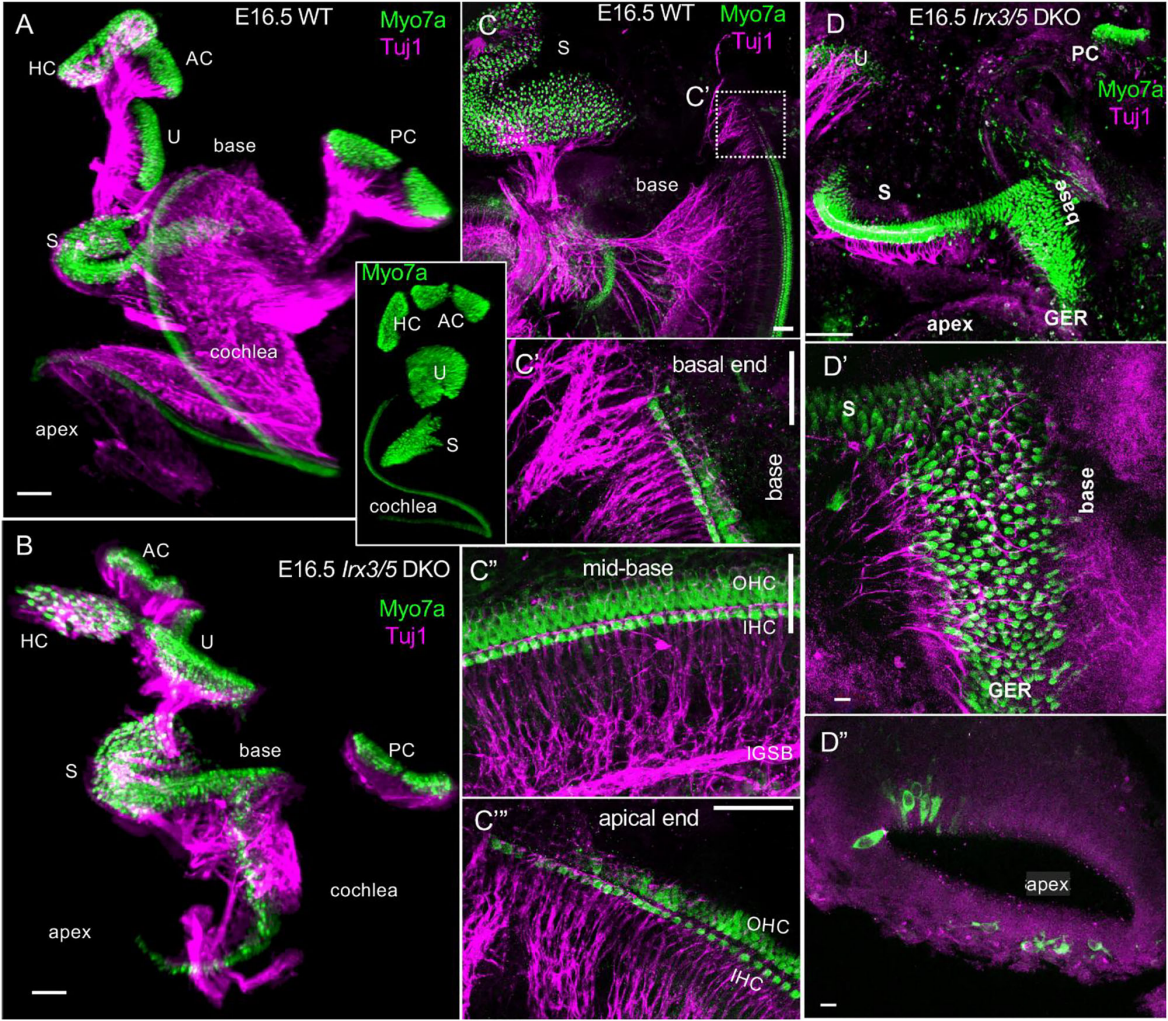
Changes in the organization of the *Irx3/5* DKO cochlea. (A, B) Light‐sheet fluorescence microscopy images of immunolabeled Myo7a (a marker of HCs) and tubulin (innervation) show the continuation of the saccule as the enlarged cochlear base in *Irx3/5* DKO, whereas the saccule is segregated from the base in the inner ear of WT mice (Videos 1 and 2), *Irx3/5* double het, *Irx3* and *Irx5*. Compared to the WT inner ear, the length of the cochlea is shortened in *Irx3/5* DKO. A very different cellular organization of the basal turn is in the cochlea of *Irx3/5* DKO (B, D) compared to WT littermates (A, C). Instead of developing a single row of inner HCs (IHCs) and three rows of outer HCs (OHCs) that will gradually reduce the number of OHCs at the end of the base of WT (C–C'''), up to ten or more vestibular‐like HCs are found in the *Irx3/5* DKO mice (D, D'). A single optical image shows an altered organization of HCs in the *Irx3/5* DKO apex with rows of HCs displaced near the modiolus (D''). The pattern of innervation in *Irx3/5* DKO mice is disorganized (B) and extends across sensory HCs in the base (D') in contrast to WT mice (A, C–C''). AC, anterior crista; GER, greater epithelial ridge; HC, horizontal crista; OC, organ of Corti; PC, posterior crista; S, saccule; U, utricle. Scale bars: 100 µm (A, B, D), 50 µm (C–C'''), and 10 µm (D'‐D'').

Typical ear development results in five vestibular sensory organs and the organ of Corti (OC) in the cochlea (Elliott et al. 2021). The three canal cristae and the utricle of *Irx3/5* DKO are comparable to the wild‐type (WT) mice (Figure 1A–C). In contrast, in the *Irx3/5* DKO, the saccule continues across the cochlea's basal turn, and the OC length is shortened by about one turn compared to 1.5 turns in the WT mice (Figure 1B,D). Instead of the single row of IHC and three rows of OHC (Figure 1A,C'–C'''; Video 1; Tables 1 and 2) in control, *Irx3/5* double het, *Irx3* KO, and *Irx5* KO (Liu et al. 2024), ten rows or more of HCs are formed in the fused cochlear base and saccule medial to the OC (Figure 1D,D'; Video 2). A single row of IHCs and three to four rows of OHCs at the apex are apparent with a displaced location at the modiolus in *Irx3/5* DKO (Figure 1D''). The tubulin‐labeled innervation of the *Irx3/5* DKO cochlea is reduced compared to WT (Figure 1A,B). Noticeably, an unusual shape of the cochlear ganglion is evident in *Irx3/5* DKO, forming two connected entities: one with a round shape near the fused end‐organ and a second, more elongated ganglion in the apex (Figure 1B). Typically, type I fibers reach the IHC, whereas type II fibers reach the first row of OHCs (Figure 1C', C''). In contrast, the *Irx3/5* DKO mice show fibers that expand across the cochlear basal turn (Figure 1D'). The apical HCs have limited innervation (Figure 1D''). Additionally, the ductus reuniens separating the saccule from the cochlear basal turn (Kopecky et al. 2012) is not formed in *Irx3/5* DKO mice (Figure S1).

**VIDEO 1:** A 3D presents an E16 WT with the five vestibular hair cells and shows four rows of cochlear hair cells labeled with Myo7a. Also labeled is the innervation, which is stained for tubulin. Note the length of the cochlea that is separate from the nearby saccule by the ductus reuniens.

**TABLE 1:** Primary antibody details.

Antibody	Host	Company	Product number and RRID	Dilution
Anti‐acetylated tubulin	Mouse	Sigma‐Aldrich	T7451, RRID:AB_609894	1:800
Anti‐myosin‐VIIa	Rabbit	Proteus Biosciences	25‐6790, RRID:AB_10015251	1:300
Anti‐NeuN	Rabbit	Cell Signaling	12943, RRID:AB_2630395	1:500
Anti‐neurofilament‐H	Chicken	MilliporeSigma	AB5539, RRID:AB_11212161	1:1000
Anti‐Pendrin	Rabbit	Bicellscientific, R and D Systems	BAF2050, RRID:AB_2143488	1:500
Anti‐collagen	Rabbit	Thermo Fisher Scientific	PA1‐26206, RRID:AB_779883	1:500

**TABLE 2:** Secondary antibody details.

Antibody	Company	Product number and RRID	Dilution
Goat anti‐chicken IgY Alexa Fluor 488	Invitrogen	A‐11039, RRID:AB_2534096	1:500
Goat anti‐mouse IgG Alexa Fluor 647	Invitrogen	A‐21235, RRID:AB_2535804	1:500
Goat anti‐rabbit IgG Alexa Fluor 488	Invitrogen	A‐11008, RRID:AB_143165	1:500
Goat anti‐rabbit IgG Alexa Fluor 546	Invitrogen	A‐11010, RRID:AB_2534077	1:500

**VIDEO 2:** The 3D of the ear of an E16.5 *Irx3/5* DKO mouse shows the four vestibular hair cells, the three canals, and the utricle. However, the saccule is fused with the basal turn. Moreover, many more hair cells can be seen in the basal turn. The ductus reuniens does not appear in the Irx3/5 DKO mouse.

**FIGURE 2:**
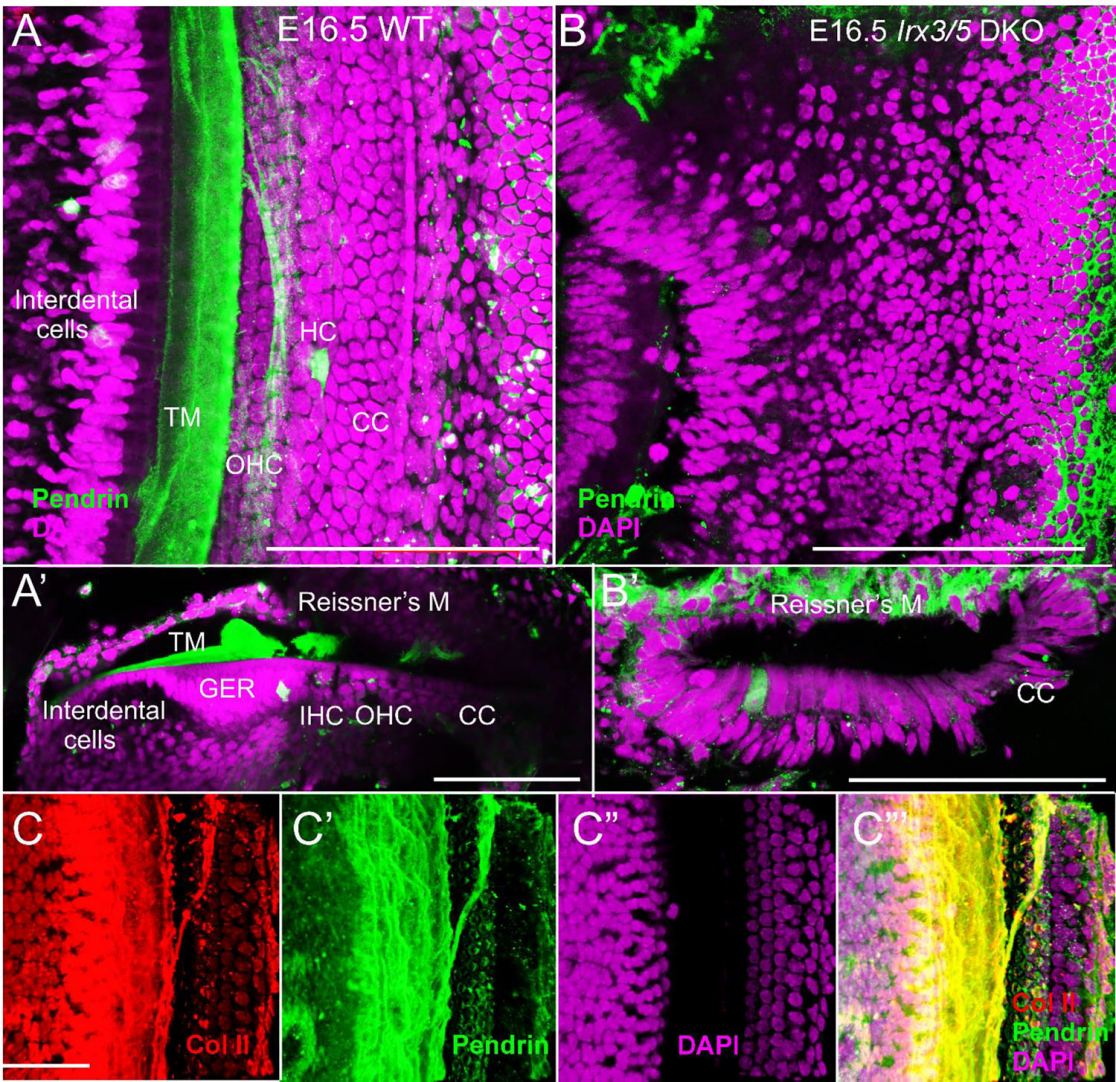
The TM is absent in the *Irx3/5* DKO mice. (A, A', C'', C''') The TM is labeled by anti‐Pendrin and anti‐Col II antibodies in WT mice. The TM overlaps with the IHC, while the TM partially covers the OHC. (B, B') The TM is absent in the cochlea of *Irx3/5* DKO mice. Note a loss of cellular organization of the cochlear epithelium in *Irx3/5* DKO. Scale bars: 100 µm (A–C).

**FIGURE 3:**
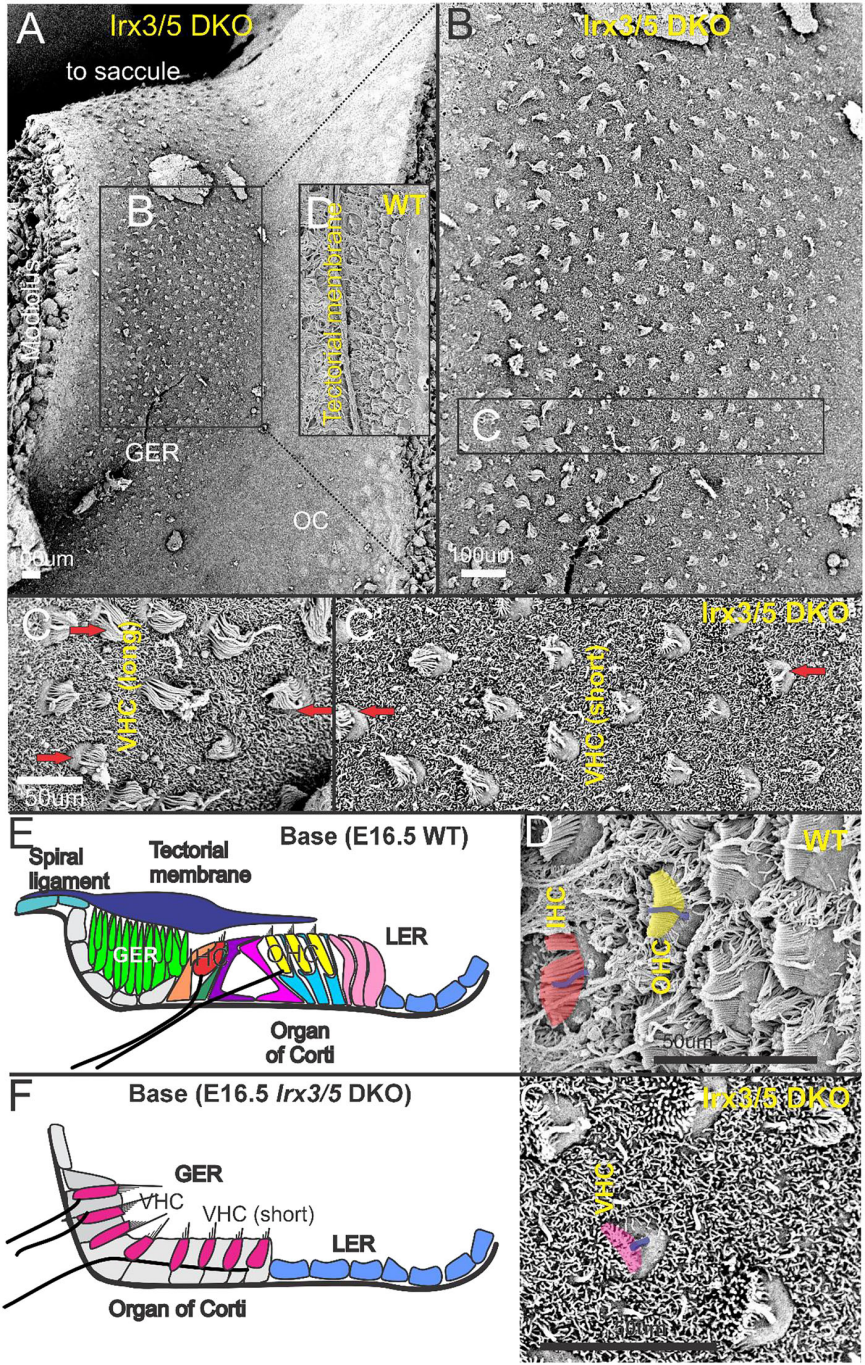
Cochlear hair cells are replaced by vestibular‐like HCs. (A) *SEM* documents the presence of 10–16 rows of HCs medial to the OC in *Irx3/5* DKO. (B) A larger magnification shows the organization of HCs with vestibular‐like HCs presented closer to the modiolus (C, C'), and cochlear‐like HCs, characterized by stereocilia and a single kinocilium (C''; lilac, blue), are formed close to the greater (GER)/lesser epithelial ridge (LER) boundary. (C, C', C'') Arrows indicate the planar cell polarity and orientation of stereocilia bundles. (D,D'). The OC of the WT cochlea is represented by a single row of stereocilia and a single kinocilium in IHC (orange, blue) and three rows of OHC (yellow, blue). (E) A schematic drawing of the WT cochlea, including the broad GER that connects with the spiral ligament to connect it with the TM. (F) In contrast, neither the TM nor the GER are formed in *Irx3/5* DKO. The cochlear epithelium is transformed into vestibular‐like HCs (VHC) closer to the modiolus, gradually transitioning into inner‐like cochlear VHCs (VHC). Note that the difference in stereocilia in IHC and OHC (D') does not fit with the shortest VHC (C''). CC, Claudius's cells; GER, greater epithelial ridge; HC, Hensen's cells; IHC, inner hair cells; OHC, outer hair cells; VHC, vestibular‐like hair cells. Scale bars: 100 µm (A, B, D) and 50 µm (C, C', C'', D').

A mixture of cochlear and vestibular‐like HCs in *Irx3/5* DKO is found in the aberrant cochlear base, which lacks TM formation. The TM starts in the spiral ligament, connects at the interdental cells (Figure 2A,A',C), and overlays HCs in the WT cochlea. In WT, anti‐Pendrin (Figure 2B) and anti‐Col II antibodies (Tables 1 and 2) show the TM (Figure 2A–C). In contrast to WT, the TM and the spiral limbus and interdental cells are absent, and cells in the greater epithelial ridge (GER) region are transformed into aberrant HCs adjacent to the modiolus in *Irx3/5* DKO (Figure 3A,B,F). The multiple rows of HCs of a fused OC‐saccule are detected in the *Irx3/5* DKO. Vestibular‐like HCs with the stereocilia arranged in bundles with centrally increasing heights are located close to the modiolus, whereas shorter HCs with linear bundles are formed close to the GER/lesser epithelial ridge (LER) boundary (Figure 3C',C''), comparable to chickens (Wu et al. 2023). Most medial HCs show a different polarity toward the modiolus, whereas much of the polarity is directed oppositely toward the GER/LER boundary (Figure 3C,C'; arrows). In normal development, the GER is attached to the TM that stretches across the inner spiral sulcus (Figure 3D,E). IHCs and OHCs in the OC in WT mice show the planar polarity of the hair bundles uniformly oriented to the abneural, lateral edge (Figure 3C',C'',D''). The development of non‐sensory cells in the cochlea of *Irx3/5* DKO is altered, resulting in the undifferentiated flat epithelium. Further work using antibodies against *Prox1* and *Sox2* for supporting cells (Fritzsch et al. 2010; Luo et al. 2021) as well as *Insm1* and *Ikzf2* to label selectively the OHC (Bi et al. 2024; Lorenzen et al. 2015; Luo et al. 2022; Sun and Liu 2023; Wang, Gu, and Liu 2024) is in progress. In addition, *Bcl11b* shows no OHC in the basal turn (Liu et al. 2024).

### 3.2 Reduced Innervation of Vestibular and Cochlear Afferents in *Irx3/5* DKO

**FIGURE 4:**
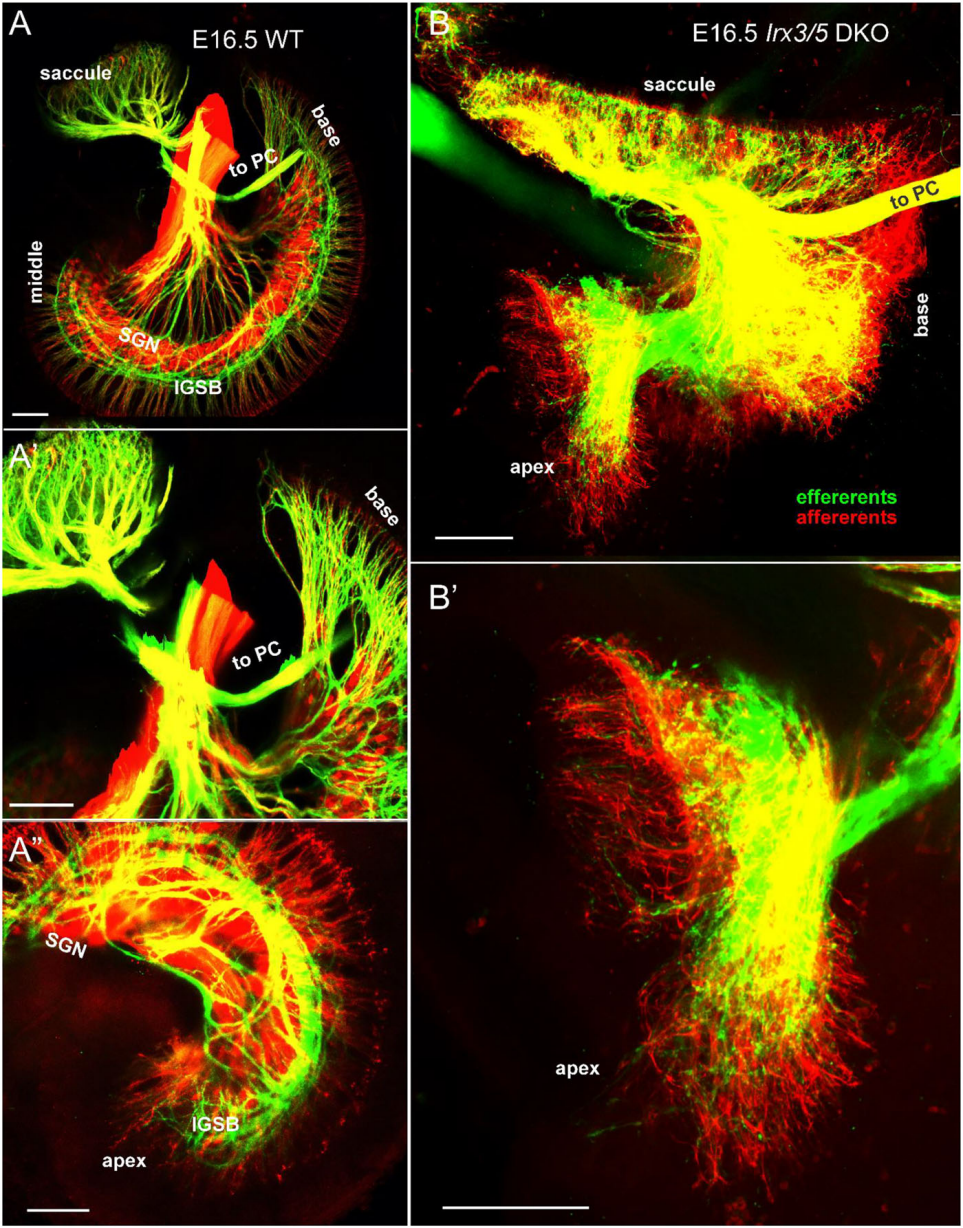
Cochlear innervation is reduced and altered in *Irx3/5* DKO. The rhombomere 4 (r4)‐applied dye (green) labels the inner ear efferents, and the r5‐applied dye (red) labels the cochlear and saccular neurons and afferent fibers. (A–A'') In the WT inner ear, the innervation of the saccule and the basal tip of the cochlea is segregated, spiral ganglion neurons (SGN) interlace with efferents form the intraganglionic spiral bundle (IGSB), radial afferent fibers reach the row of inner HCs. (B, B') In *Irx3/5* DKO, the innervation of the saccule and the cochlear base is fused, and cochlear neurons form a round‐shaped ganglion connected to a more elongated ganglion in the apical part of the shortened cochlea. Afferent and efferent fibers overlap, the IGSB is not formed, and the radial afferent fibers are short and widely ramified. Note the posterior canal (PC) fibers exit close to the saccule and are comparable between WT and *Irx3/5* DKO. Scale bars: 100 µm.

Afferent and efferent nerve fibers extending to the six sensory epithelia of the inner ear can be traced using lipophilic dyes (Figure 4). IEE can be traced selectively with dye inserted ventrally in the rhombomere 4, which labels the vestibular HCs and the intraganglionic spiral bundle (IGSB, green; Figure 4A,B; Figure S2). A more dorsal insertion of different dyes into rhombomere 5 labels the SGNs (red; Figure 4A,B) and vestibular ganglion neurons (VGNs; Figure S2). Overall, the pattern of innervation is similar in the three canal cristae and the utricle between WT and *Irx3/5* DKO mice (Figure S2). In contrast, the innervation pattern of the saccule and cochlea is different in *Irx3/5* DKO mice. The WT saccule is densely innervated and segregated from the cochlea, with SGNs forming the spiral ganglion wrapped by IGSB (Figure 4 A–A''). Both afferents and efferents form radial fibers that innervate the IHCs, with a few fibers reaching the first row of OHCs in the cochlear base (Figure 4A'; Figure S2). In *Irx3/5* DKO mice, the caudal saccular innervation blends into the tip of the basal turn without distinct separation, resulting in broad innervation of the basal turn connected to an unusual round‐shaped ganglion at the cochlear base (Figure 4B). The second ganglion is recognizable at the area of the cochlear apex (Figure 4B,B'). Afferents and efferents overlap, and no formation of the IGSB is found in the shortened cochlea of *Irx3/5* DKO. The IEE fibers reach a much shorter distance from the OC in the *Irx3/5* DKO than the WT (Figure 4A'',B'; Figure S2). The WT mice show lengthy radial fibers barely reaching the row of IHCs. In contrast, efferent and afferent fibers ramify wildly in the *Irx3*.5 DKO (Figure 4B,B').

### 3.3 Vestibular and Auditory Neuronal Connectivity in the *Irx3/5* DKO and WT Inner Ear

**FIGURE 5:**
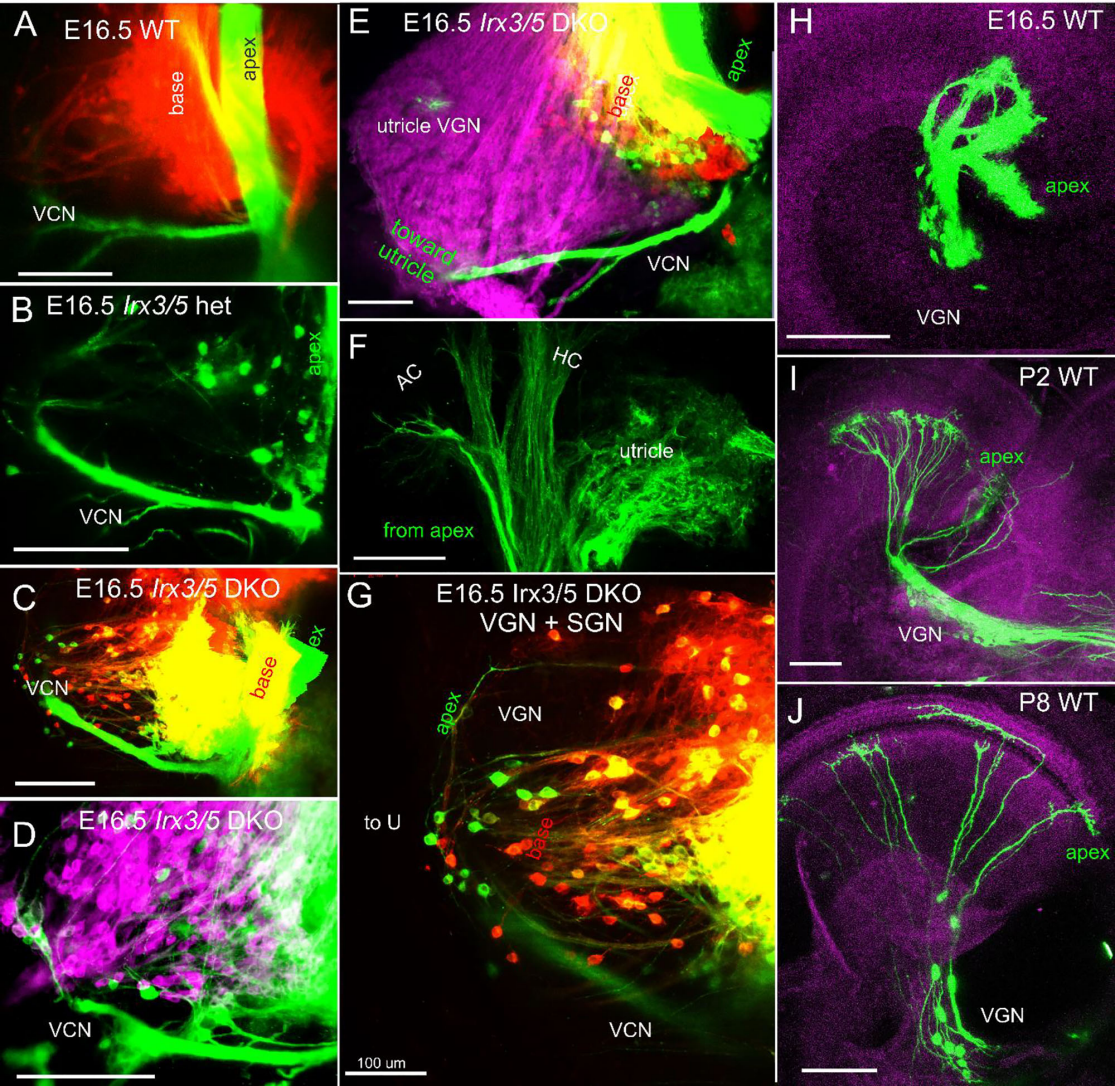
Vestibular and cochlear neurons are mingled in *Irx3/5* DKO, an unknown connection between the apex and the vestibular system. (A, B) Dye applications to the base (red) and apex (green) label corresponding spiral ganglion neurons and fibers in WT and *Irx3/5* heterozygous. In contrast, cochlear neurons and their connections with vestibular ganglion neurons can be traced in *Irx3/5* DKO (C–E, G). An unknown “vestibular‐cochlear” nerve (VCN) exists in all WT, *Irx3/5* het, and *Irx3/5* DKO mice (A–E, G). (F) These fibers project directly from the cochlear apex to the utricle and the anterior and horizontal canal (AC, HC). (H–J) A corresponding unique population of neurons and apical fibers is labeled by dye insertion into the VCN (green) and is located near the apex in WT mice. Scale bars: 100 µm.

Dye insertions in rhombomeres 4 and 5, visualizing afferents and inner ear neurons (red) and efferents (IEE and facial nerve, green), demonstrate a diminished superior and inferior vestibular ganglion size in *Irx3/5* DKO compared to WT (Figure S3A,B,D,E). A shorter and unusually shaped cochlea is delineated by cochlear IEE fibers (Figure S3D). The aberrant shape of the spiral ganglion in the *Irx3/5* DKO cochlea is also shown by dye tracing of afferents, revealing a round‐shaped ganglion formation connecting the base and saccule and a more linear‐shaped ganglion in the apex (Figure S3C,F,F'). Dye insertions into the apex (green), base (red), and saccule/utricle (lilac) label VGNs and SGNs and central axons of the auditory and vestibular nerves (Figure 5). In *Irx3/5* DKO, dye‐labeled cochlear fibers overlap, indicating a loss of apical and basal spatial segregation of SGNs compared to WT (Figure 5A,C,E). Additionally, some SGNs colocalize with VGNs in *Irx3/5* DKO, indicating the formation of an unsegregated aberrant ganglion (Figure 5C,D,G).

We show a novel interconnection between the cochlear apex and the vestibular organs, a “vestibular‐cochlear” nerve (VCN), shown by dye application to the apex (Figure 5A–G). This unique connection was found in all WT, *Irx3/5* heterozygous, and *Irx3/5* DKO mice. The apical tip dye tracing labeled between 4 and 20 ganglion neurons, representing a unique set of neurons near the apex and below the vestibular ganglion. To further investigate this unique vestibular‐cochlear connection, the dye was selectively inserted at the tip of the VGN, indicated by VCN (Figure 5A), in WT mice at E16.5, P2, and P8 (Figure 5H–J). About 30–50 ganglion neurons are labeled, exclusively ramifying at the tip of the apex. These “vestibular‐cochlear” neurons interconnect the vestibular organs, the utricle, and the anterior and horizontal cristae with the cochlear apex (Figure 5F) and form the “vestibular‐cochlear” ganglion in embryos and postnatal mice (Figure 5H–J).

### 3.4 Cochlear Fibers Overlap With Vestibular Fibers to Reach the Auditory and Vestibular Nuclei in *Irx3/5* DKO

**FIGURE 6:**
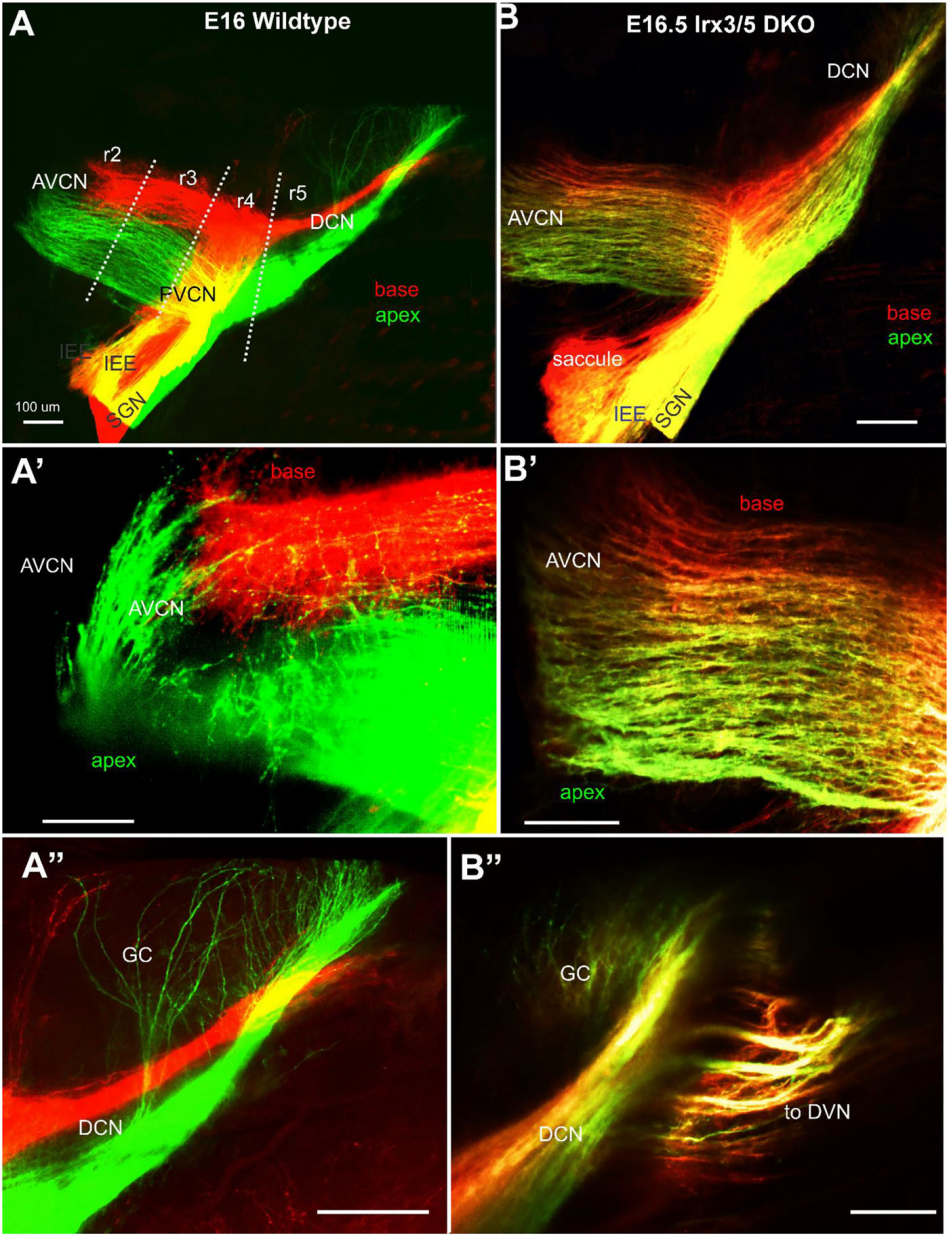
Central projections are incompletely segregated in the cochlear nuclei of *Irx3/5* DKO. (A‐A'') Dye tracing labeling the central projections from the cochlear apex (green) and base (red) shows the segregation of fibers in the cochlear nucleus of WT mice (the anteroventral cochlear nucleus [AVCN] and dorsal cochlear nucleus [DCN]). (A'') Note a few fibers that innervate the granular cells (GC). (B–B'') In *Irx3/5* DKO mice, the central projections from the base and the apex show an unusual overlap with the graded labeling of fibers. A unique caudal projection from the DCN expands toward the descending vestibular nuclei (DVN), interconnecting the auditory and vestibular systems. Scale bars: 100 µm.

We evaluated the auditory nerve fiber projections to the brainstem cochlear nucleus (CN) complex, composed of the anterior ventral CN (AVCN), posterior ventral CN (PVCN), and dorsal CN (DCN). Dye tracing from the cochlear base (red) and apex (green) reveals the typical segregation of innervation and the tonotopic organization in the CN of WT mice (Figure 6A) (Elliott et al. 2023; Filova, Bohuslavova, et al. 2022; Filova, Pysanenko, et al. 2022; Macova et al. 2019). In contrast, in *Irx3/5* DKO, dye tracing shows an intercalation between the apex (more ventral) and the base projections (more dorsal; Figure 6B,B') that ramifies to interact with the dorsal fibers (Figure 6A'). The graded labeling of fibers from the apex and base in *Irx3/5* DKO mice is unique, suggesting an altered tonotopic organization. A noticeably shorter rostral projection from the base and the apex in the AVCN is found in *Irx3/5* DKO (Figure S4). Apical fibers that reach the length of ∼420 µm in the WT are shortened to ∼330 µm in *Irx3/5* DKO (Figure S4). An aberrant connection of the cochlea with the vestibular system in *Irx3/5* DKO is shown by either apex or base dye‐labeled fibers reaching out from the DCN to the descending vestibular nuclei (DVN; Figure 6B''). No projections from cochlear dye tracing to the DVN (Maklad and Fritzsch 2002) are labeled in WT mice (Figure 6A''). Fibers mainly from the apex, but a few fibers from the base, innervating the granular neurons (GC) in the superficial layer of the DCN of WT mice and to a lesser extent in *Irx3/5* DKO (Figure 6A'',B'').

**FIGURE 7:**
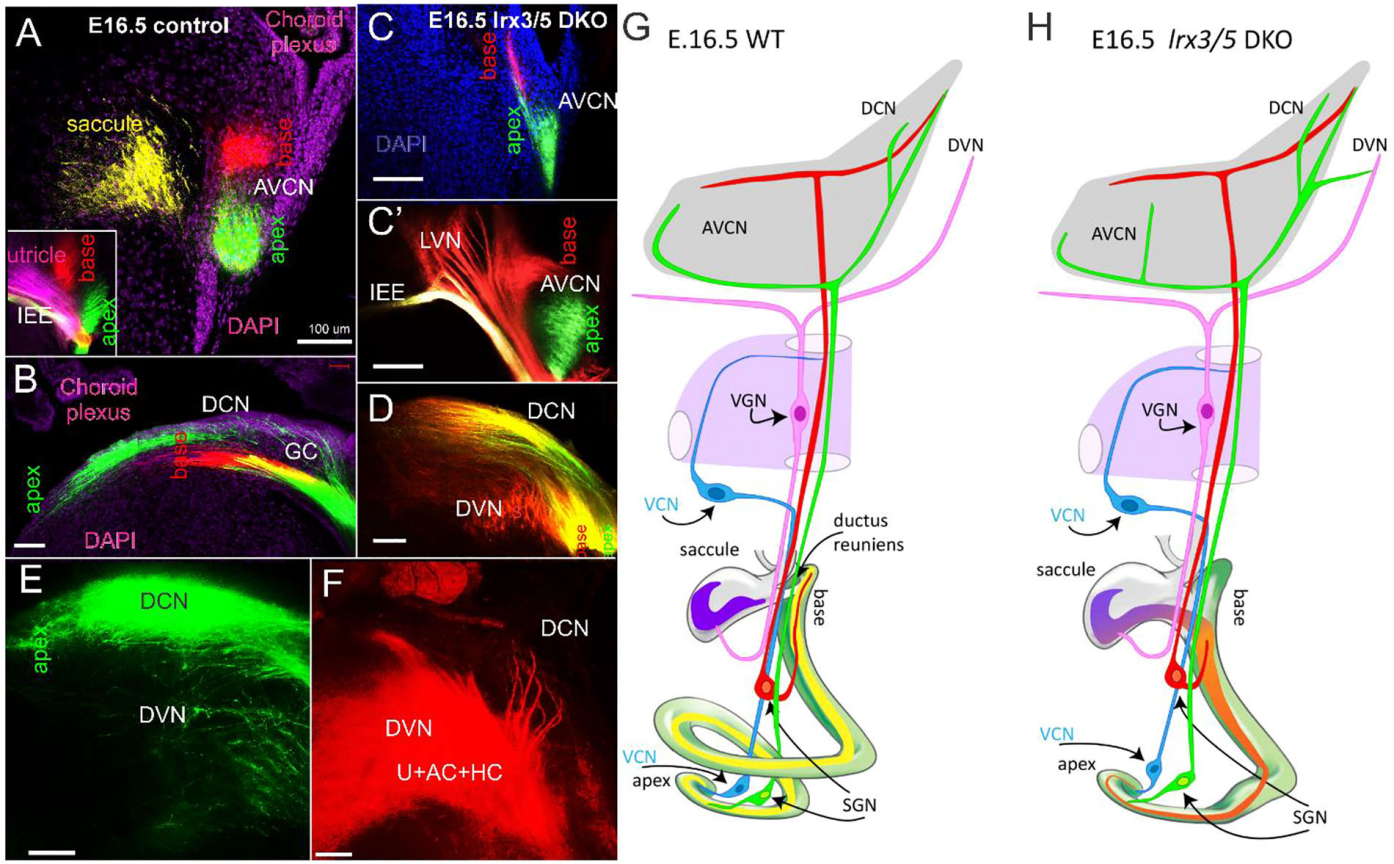
The cochlear and vestibular nuclei are interconnected in the brainstem of *Irx3/5* DKO. (A, B) The central projections from the apex, base, and saccule are segregated in WT mice. Likewise, the central projections from the utricle, base, and apex are labeled separately (insert in A). (C, C') In *Irx3/5* DKO, the entry near the IEE shows that fibers labeled from the fused base‐saccule end‐organ innervate the lateral vestibular nuclei (LVN) and broadly overlap with extensions to the anteroventral cochlear nucleus (AVCN). (D) Similarly, the central projections from the base and apex to the dorsal cochlear nucleus (DCN) overlap, and branches labeled by the base‐dye application reach the descending vestibular nuclei (DVN) of *Irx3/5* DKO. (E) The apical dye tracing of fibers from the apex shows the DCN fibers that branch to innervate the DVN. (F) The dye applications to the vestibular end‐organs (U, AC, HC) label the central projections to the DVN and the DCN, interconnecting the vestibular and cochlear nuclei of *Irx3/5* DKO. (G) In WT mice, the cochlea has 1.5 turns, and the saccule is spatially segregated from the cochlear base by the ductus reuniens. The central projections from the base and apex to the cochlear nuclei are segregated. (H) In *Irx3/5* DKO, the cochlea is shortened to one turn, and the saccule continuously transforms into the base, which is innervated by spiral ganglion neurons (SGN) and vestibular ganglion neurons (VGN). The cochlear afferents from the base and apex are unsegregated, forming interconnections to the descending vestibular nuclei (DVN). A novel type of neuron, the vestibulo‐cochlear neurons (VCN), connect the apex with VGN. AC, anterior canal; DAPI, nuclei staining; GC, granule cells; HC, horizontal canal; U, utricle; Bar indicates 100 µm.

We investigated the interconnecting central projections between the cochlear and vestibular nuclei in the brainstem of *Irx3/5* DKO. The central projections reaching the cochlear and vestibular nuclei are labeled in three distinct colors, allowing differentiation between the apex, base, and saccule (Figure 7A) or the utricle (insert in Figure 7A) in WT mice. Compared to a large gap shown between the apex‐ and base‐labeled fibers in WT mice (Figure 7A, insert), incomplete fiber segregation from the base and apex is found in the *Irx3/5* DKO brainstem (Figure 7C). Because of the base‐saccule fusion in *Irx3/5* DKO, the base‐inserted dye co‐labels fibers reaching the lateral vestibular nuclei (LVN; Figure 7C'). There is also a massive overlap between the apex and base that expands in the DCN of *Irx3/5* DKO mice (Figure 7D) compared to WT mice (Figure 7B). Both lipophilic dyes applied to the apex and base show labeled fibers in the DVN of *Irx3/5* DKO (Figure 7D,E), whereas no fibers are labeled in the DVN of WT littermates (Figure 7B). Similarly, dye applications into the utricle, anterior canal, and horizontal canal label fibers that extend from the DVN into the DCN of *Irx3/5* DKO (Figure 7F), revealing an aberrant central interconnection between cochlear and vestibular nuclei. In summary, central projections from the cochlea lack segregation between the apex and the base in *Irx3/5* DKO mice (Figure 7G,H).

## 4 Discussion

Analyses of *Irx3/5* DKO reveal several distinctive features in inner ear development. First, the deletion of *Irx3/5* leads to the formation of a unique end‐organ resulting from the fusion of the cochlear basal turn and the saccule, where vestibular HCs gradually transform into cochlear HCs (Figures 1, 2, 3). Non‐sensory cell development is altered, resulting in an undifferentiated flat epithelium and the absence of otoconia and TM in the transformed cochlea. Second, cochlear neurons form two distinctly shaped ganglia (Figures 1 and 4) and project central blended fibers from the apex and the base (Figures 6 and 7). Third, the auditory and vestibular systems of *Irx3/5* DKO are interconnected, characterized by the formation of bilateral connections between the DVN and DCN. Additionally, we show a unique interconnection between the cochlear apex and the vestibular organs, a “vestibular‐cochlear” nerve (VCN) that exists in the mouse inner ear (Figures 4 and 6) and provide an interpretation for the absent lagena in mammals (Figure S5).

### 4.1 *Irx3* and *Irx5* Are Required for Non‐Sensory Cell Development in the Inner Ear

We document that the covering of the maculae (otoconial membrane) of the saccule and the OC TM are absent in the *Irx3/5* DKO mice, as shown by Pendrin (Koh et al. 2023) and collagen immunolabeling and *SEM* (Figure 2). Most apparent is the transformation of the cells of the GER, typically producing collagens and glycoproteins of the TM, including α‐ and β‐*tectorin* and *Emilin2* (Jean et al. 2023; Pressé, Malgrange, and Delacroix 2023), which connects with the HC bundle‐tips (Figure 1, 2). The TM, a ribbon‐like strip of extracellular matrix, is attached to the surface of the spiral limbus, spans the interdental cells, and overlays the sensory epithelium, connecting to the OHCs' hair bundle tips (Figure 3; Goodyear and Richardson 2018). Non‐sensory supporting cells expressed genes encoding *otogelin* necessary for the formation of the TM attachment to the OHC bundles; *otoancorin* produced by the interdental cells of the spiral limbus is essential for the attachment of the TM to the spiral limbus (de Sousa Lobo Ferreira Querido et al. 2023; Goodyear et al. 2017; Jean et al. 2023; Mulry and Parham 2020). Interestingly, *Irx3‐* and *Irx5*‐deficient mouse models showed craniofacial abnormalities and reduced expression of the osteogenic regulators and osteoblastic‐specific markers, such as collagen Types II, IV, IX, and X, indicating changes in the cell fate of mesenchymal stem cells (Jiang et al. 2022; Li et al. 2014; Narwidina et al. 2023; Tao et al. 2020). For example, *IRX3* and *IRX5*, downstream members of the Wnt signaling pathway, regulate osteogenic lineage commitment (Tan et al. 2020). Deletion of *Irx3*/*Irx5* has been shown to increase *Shh* signaling and disrupt the formation of anterior hindlimb progenitors (Li et al. 2014). Additionally, osteoblast lineage‐specific deletion of *Irx3* in *Irx5* ^−/−^ mice impaired the expression of mineralization genes and craniofacial defects (Cain et al. 2016). Results indicate that *Irx3/5* deletion alters the differentiation of two regions of the cochlear duct, which is crucial for the development of OC GER. Mainly, epithelial supporting cells originating from the GER, including the spiral sulcus and limbus cells, are absent, and the TM and otoconia membranes are not formed in *Irx3*/*Irx5* DKO mice. *Irx3/5* deletion expands sensory HCs at the expense of other epithelium cell types, suggesting that *IRX3* and *IRX5* are required for correct non‐sensory cell fate within the cochlear duct epithelium. Moreover, the organization of long and short HCs in *Irx3/5* DKO mice resembles that in birds (Wu et al. 2023).

The ductus reuniens normally segregates from the basal turn of the cochlear and saccule (Kopecky et al. 2012), like *Irx3/5* double het, *Irx3*, and *Irx5* null mutations (Liu et al. 2024). During ear development, the non‐sensory constrictions and the formation of the substantial thinning of the spaces between the saccule and cochlear duct as the presumptive ductus reuniens becomes prominent at E16.6 (Kopecky et al. 2012). Moreover, the saccule in *nMyc* null mice has distinct cochlear and vestibular HCs (Kopecky et al. 2011). A similar but more profound segregation cue disruption in inner ear development has been reported without *Lmx1a* (Nichols et al. 2008). *Lmx1a* mutant mice have only three sensory epithelia in the inner ear: two enlarged canal cristae and one fused utriculo‐sacculo‐cochlear sensory epithelium comprising an amalgamation of the cochlea, saccule, and utricle. In the fused end‐organ, the cochlear basal region is distinguished by the presence of a TM and cochlea‐specific innervation, and the cochlea‐like apex displays minor disorganization of the HCs and supporting cells (Nichols et al. 2008). Moreover, overexpression of *Atoh1* (Zheng and Gao 2000) and absence of *Neurog1* (Matei et al. 2005) can result in additional hair cells in the GER. Misexpression of *Neurog1* can partially substitute for *Atoh1* (Jahan et al. 2015) that likely adds downstream of *Tbx2* (Bi et al. 2024).

In contrast, *Irx3/5* DKO mice show a unique transformation that shows a gradual change from vestibular HCs, from the modiolus with the GER to the basal region of the cochlea (Figures 3 and 4). Additionally, the TM and the adjacent saccular otoconia are absent (Figure 2). In summary, the deletion of *Irx3/5* results in an expansion of sensory HCs at the expense of non‐sensory cell types. The result suggests that *Irx3* and *Irx5* are required for non‐sensory development within the cochlear duct epithelium and for cochlear‐saccular segregation cues.

### 4.2 *Irx3/5* DKO Affects Inner Ear Neurons and Their Peripheral and Central Projections

We report and define a novel interconnection between the cochlear apex and the vestibular organs, a “vestibular‐cochlear” nerve (VCN), as shown by dye tracing (Figure 5A–G). This unique neuronal projection from the apex to the utricle, the anterior, and the horizontal canal was found in all WT, *Irx3/5* heterozygous, and *Irx3/5* DKO mice. A similar connection was reported in the *Foxg1* KO mice (Pauley, Lai, and Fritzsch 2006), *Neurod1* KO mice (Filova, Bohuslavova, et al. 2022; Macova et al. 2019), and in control and *Npr2* KO mice in E16.5 and 18.5 that was referred to as “cochlear‐vestibular anastomosis” (CVA) (Schmidt and Fritzsch 2019). We suggest referring to neurons that form this unique connection between the apical end and the vestibular organs as the vestibulo‐cochlear neurons (VCN).

A distinct interconnection between the vestibular and auditory systems is found only in the *Irx3/5* DKO mice. The fusion of the saccule and the cochlear base in the *Irx3/5* DKO results in a loss of separation between the efferents and afferents and between vestibular and cochlear innervation. Centrally, a distinct reciprocal interaction exists between cochlear and vestibular nuclei, evidenced by branches extending from the DCN to the DVN and from the DVN to the DCN (Figures 6 and 7). The unique interconnection between the DCN and DVN requires viable *Irx3/5* DKO mice to comprehend the physiological significance.

### 4.3 *Irx3/5* DKO Mice Show a Limited Tonotopic Organization

Central projections segregated into distinct base, middle, and apex are necessary for organized tonotopic cells (Pyott et al. 2024). Previous work showed disorganization of central projections in specific mutations, resulting in dysfunctional tonotopy of the auditory processing (Filova, Bohuslavova, et al. 2022; Filova, Pysanenko, et al. 2022; Macova et al. 2019). Although the *Irx3/5* DKO mice demonstrate a near‐normal development of CN, the graded central projections with blended fibers from the apex and base indicate a disorganized primary tonotopic auditory map (Figure 6). The investigation of the unique graded projection effects on higher order tonotopic information processing in the auditory system is hindered by the embryonic lethality of the *Irx3/5* DKO mutation.

### 4.4 *Irx3/5* Likely Plays a Role in the Origin of Lagena and Basilar Papilla

*Irx3/5* are present from flies to mammals (Cardeña‐Núñez et al. 2017; Kerner et al. 2009; Li et al. 2014). A notable duplication results in pairs in *Irx1/3, Irx2/5*, and *Irx4/6*. Moreover, *Irx3* and *Irx5* are unique in that they segregate between the hagfish/lampreys, sharks, teleosts, coelacanth, amphibians, and amniotes that split from each other about 440 to 300 million years ago (Marlétaz et al. 2024; Yu et al. 2024). Interactions between ear development and the segregation of saccule, basilar papilla, and lagena are incompletely understood (Liu et al. 2024). What is known is that the lagena forms at least three times independently in elasmobranchs, modern teleosts, and sarcopterygians but is lost in the mammalian cochlea (Figure S5). The basilar papilla is formed in monotremes, which likely transform into the lagena in mammals (Fritzsch, Schultze, and Elliott 2023; Schultz, Zeller, and Luo 2017). Interestingly, the *Irx3/5* DKO saccule and basal turn [equal to the basilar papilla] are a continuum from vestibular to likely IHCs. Compared to *Irx3/5* DKO mice, one would assume that minor variations of the basilar membrane can result in the formation of the TM and HCs complexity (Fritzsch and Elliott 2023; Liu et al. 2024; Manley 2023), which is, after all, a separate sensory organ found only in most sarcopterygians. The basilar papilla (or cochlea in derived mammals) connects with the TM in *Latimeria*, most amphibians, and amniotes that resembles *Irx3/5* DKO that only develops short and long cilia, like in chickens (Wu et al. 2023). The connection between the apex (or lagena) and the utricle requires additional studies.

## Author Contributions

All authors listed have made a substantial, direct, and intellectual contribution to the work and approved it for publication.

### Peer Review

The peer review history for this article is available at https://publons.com/publon/10.1002/cne.70008.

## Supporting information



Figure S1. The ductus reuniens separating the saccule and the cochlear base is not formed in *Irx3/5* DKO.
Figure S2. Innervation in the ear of *Irx3/5* DKO is reduced and altered.
Figure S3. Dye labeling in the brainstem shows differences in the innervation of the *Irx3/5* DKO inner ear.
Figure S4. Central cochlear afferents are shorter and unsegregated in the cochlear nucleus of *Irx3/5* DKO compared to their WT littermates.
Figure S5. A remarkable parallel exists between all vertebrates regarding *Irx3* and *Irx5*.

## Data Availability

The original contributions presented in the study are included in the article. Further inquiries can be directed to the corresponding author. The data that supports the findings of this study are available in the supplementary material of this article.
