# Supplementary material for: Irx3/5 Null Deletion in Mice Blocks Cochlea‐Saccule Segregation and Disrupts the Auditory Tonotopic Map
Source: J Comp Neurol. 2024 Dec 10;532(12):e70008. doi: 10.1002/cne.70008 (PMC11629443; doi:10.1002/cne.70008)
Supplement: Supplementary file 1 — Figure S1. The ductus reuniens separating the saccule and the cochlear base is not formed in Irx3/5 DKO. Figure S2. Innervation in the ear of Irx3/5 DKO is reduced and altered. Figure S3. Dye labeling in the brainstem shows differences in the innervation of the Irx3/5 DKO inner ear. Figure S4. Central cochlear afferents are shorter and unsegregated in the cochlear nucleus of Irx3/5 DKO compared to their WT littermates. Figure S5. A remarkable parallel exists between all vertebrates regarding Irx3 and Irx5. [file CNE-532-e70008-s001.docx]

SI Appendix:


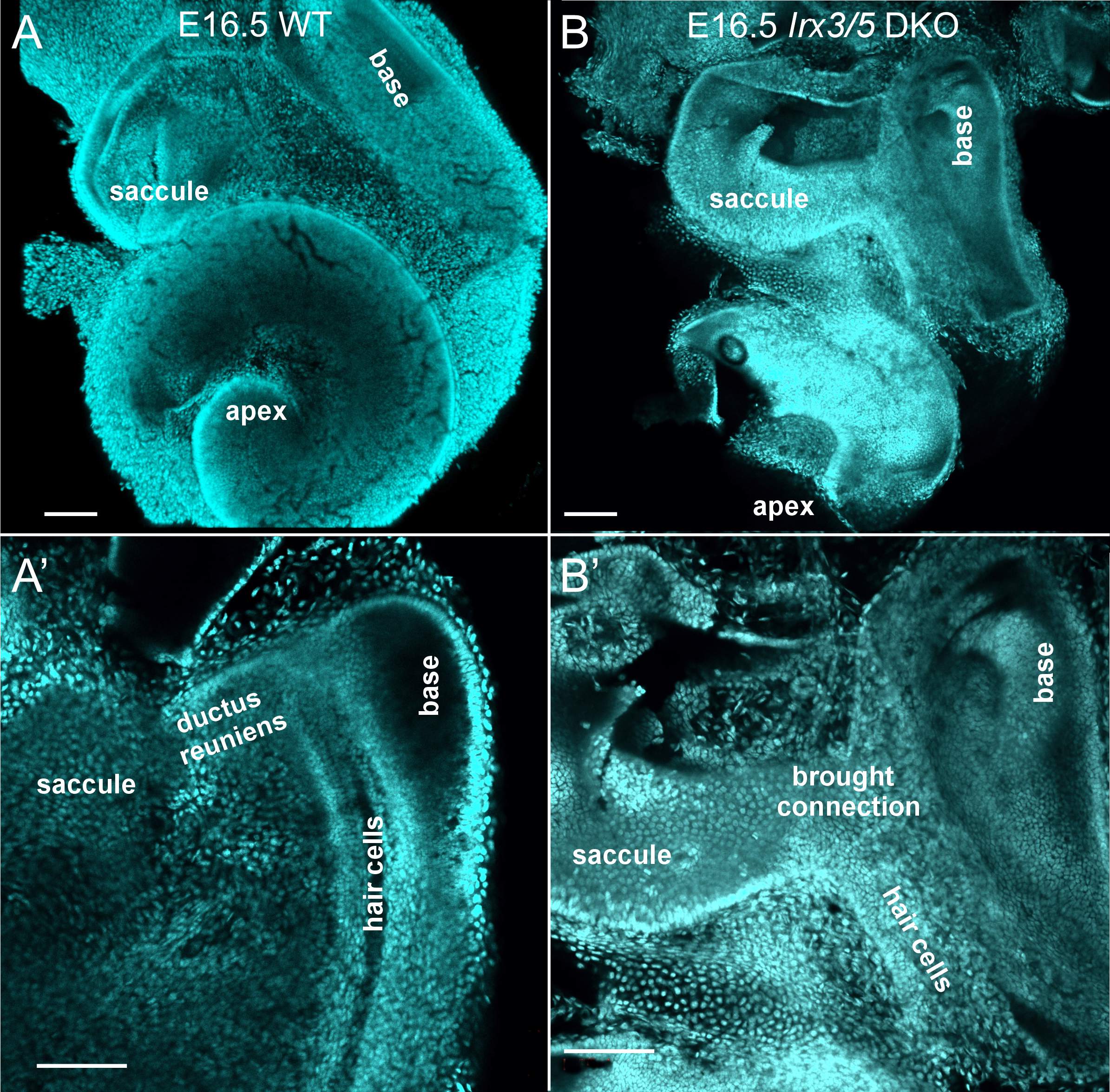


Fig. S1. The ductus reuniens separating the saccule and the cochlear base is not formed in *Irx3/5* DKO. (A, A’) The ductus reuniens separated the saccule and the basal turn in the WT inner ear. (B, B’) The cochlear base and saccule are merged in *Irx3/5* DKO mice. Note the shortened one-turn cochlea in mutants. Scale bars: 100 µm.

Fig. S2. Innervation in the ear of *Irx3/5* DKO is reduced and altered. (A, B) The main difference between the WT and mutant inner ear is the cochlea and saccule (S) innervation pattern. Note the difference in the shape of the spiral ganglion and the ramification of cochlear efferents and afferents, with the intraganglionic spiral bundle (IGSB) absent in the *Irx3/5* DKO cochlea. (A’, A”, B’, B”) In contrast, the utricle, anterior, horizontal, and posterior canal (U, AC, HC, PC) are present in WT and *Irx3/5* DKO mice. However, the fiber length and width are reduced in the mutant inner ear compared to WT mice. Scale bars: 100 µm.


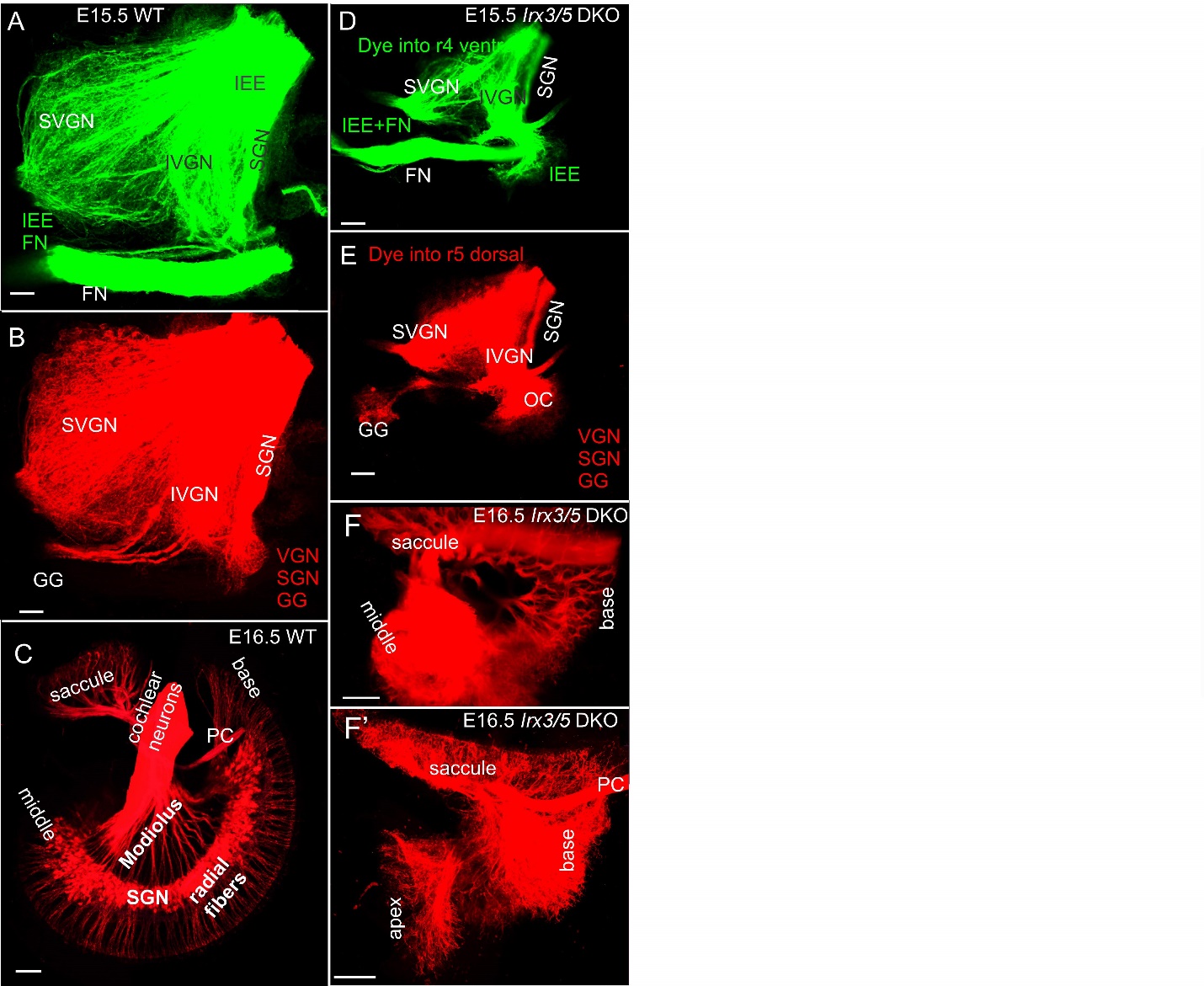


Fig. S3. Dye labeling in the brainstem shows differences in the innervation of the *Irx3/5* DKO inner ear. (A, D) Dye applications into the rhombomere 4 ventral (green) labeling inner ear efferents (IEE) and facial nerve (FN) show that the superior and the inferior vestibular ganglia (SVGN, IVGN) and IEEs are reduced in mutant compared to WT mice. (B-F’) Dye applications into the rhombomere 4 dorsal (red) labeling inner ear afferents and neurons show reduced inner ear ganglia and disorganized cochlear afferent innervation in *Irx3/5* DKO compared to the WT cochlea with the characteristic radial fibers and distinctive innervation of the saccule and the base. Note, in *Irx3/5* DKO, the unusual shape of the cochlear ganglion and neurons directly adjacent to the HCs (F, F’). Scale bars: 100 µm.

Fig. S4**.** Central cochlear afferents are shorter and unsegregated in the cochlear nucleus of *Irx3/5* DKO compared to their WT littermates. The *Irx3/5* DKO mice have a shorter rostral projection from the base (230 µm versus 300 µm in WT) and even shorter ventral apex fibers (330 µm versus 420 µm in WT), as shown by dye tracing. AVCN, anteroventral cochlear nucleus; DCN, dorsal cochlear nucleus. Scale bars: 100 µm.


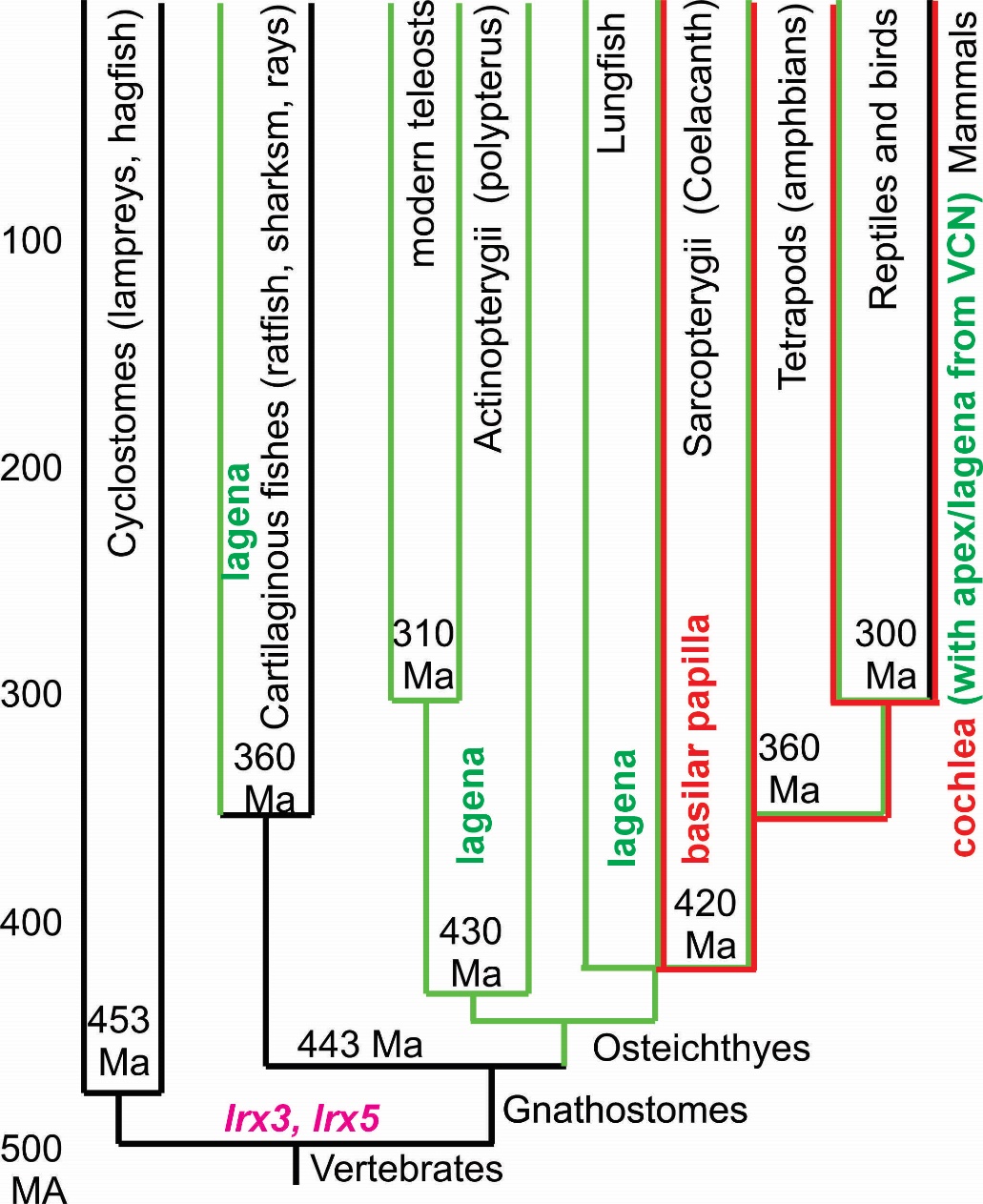


Fig. S5. A remarkable parallel exists between all vertebrates regarding *Irx3* and *Irx5*  (<https://useast.ensembl.org/Homo_sapiens/Gene/Compara_Tree?db=core;g=ENSG00000176842;r=16:54930865-54934485;collapse=19354469,19354657,19354551,19354517,19354475,19354480,19354521,19354559,19354553,19354499,19354432,19354665,19354666,19354341>). The same split into vertebrates following the same dendrogram: hagfish>sharks>bony fish>coelacanth>amphibians>amniotes (see data presented above). We had a limited set of ear expressions in chickens and mice. It would be essential to expand the *Irx3/5* expression in crucial vertebrates that lack a saccule, lagena in cyclostomes, which have a parallel evolution in derived sharks and modern teleosts, split as a separate lagena (that evolved three times independently, in green line), while coelacanth, amphibians, and amniotes split a new organ, the basilar papilla (red line), that likely depends on *Irx3/5* shown in the fusion of saccule and basal turn of the cochlea in *Irx3/5* DKO mice.
